# Effect of NaCl on the Rheological, Structural, and Gelling Properties of Walnut Protein Isolate-κ-Carrageenan Composite Gels

**DOI:** 10.3390/gels8050259

**Published:** 2022-04-21

**Authors:** Yuqing Lei, Hui Ouyang, Wu Peng, Xiongwei Yu, Long Jin, Shugang Li

**Affiliations:** 1 Key Laboratory for Agricultural Products Processing of Anhui Province, Engineering Research Center of Bioprocess, School of Food and Biological Engineering, Hefei University of Technology, Ministry of Education, Hefei 230601, China; leiyuqing2022@163.com (Y.L.); ouyanghui1994@163.com (H.O.); pengwu0313@163.com (W.P.); 2 Key Laboratory of Fermentation Engineering, School of Food and Biological Engineering, Hubei University of Technology, Ministry of Education, Wuhan 430068, China; 3 Wuhan Xudong Food Co., Ltd., Wuhan 430040, China; yuxiongwei321@163.com; 4 Qiaqia Food Co., Ltd., Hefei 230601, China; jinlong@qiaqiafood.com

**Keywords:** NaCl, walnut isolate protein, κ-carrageenan, gel characteristics

## Abstract

In this study, we discovered that a certain concentration of Na^+^ (15 mM) significantly improved the bond strength (12.94 ± 0.93 MPa), thermal stability (72.68 °C), rheological properties, and textural attributes of walnut protein isolate (WNPI)-κ-carrageenan (KC) composite gel. Electrostatic force, hydrophobic interaction, hydrogen bond, and disulfide bond were also significantly strengthened; the α-helix decreased, and the β-sheet increased in the secondary structure, indicating that the protein molecules in the gel system aggregated in an orderly manner, which led to a much denser and more uniform gel network as well as improved water-holding capacity. In this experimental research, we developed a new type of walnut protein gel that could provide technical support for the high-value utilization and quality control of walnut protein.

## 1. Introduction

Walnut (*Juglans regi L.*) is a member of the Juglandaceae family that is particularly popular among consumers for its rich nutritional value [1,2]. China is the world’s largest producer and consumer of walnuts, and most walnuts are used for oil extraction due to their extremely high content of polyunsaturated fatty acids [3]. Walnut meal is a byproduct of walnut oil production. Studies have shown that walnut meal contains more than 50% protein, but it is usually discarded, resulting in a waste of walnut protein [4]. In addition, walnut protein can be added to functional food as a natural raw material with good functionals characteristics and biological activity, which plays an important role in promoting human health [5]. Glutelin is the main protein group present in walnut protein (about 70%), and its low water solubility limits the functional performance of walnut protein in food [6]. At present, research on walnut protein is mainly focused on protein components, extraction and preparation methods, physical and chemical properties, and biologically active peptides [7,8,9,10]. There are few reports on gel and adhesion properties of walnut protein. Therefore, improving the gel properties of walnut protein is very important for the development of new gel foods for various applications.

Protein-polysaccharide complexes are commonly used to enhance the physicochemical properties of proteins [11]. Studies have shown that the electrostatic interactions, hydrophobic interactions, hydrogen bonds, and disulfide bonds between proteins and polysaccharides can overcome the repulsion between molecules and improve the final performance of the gel [12,13]. In addition, the filling effect of polysaccharides is conducive to the further cross linking of protein chains during heat treatment [14]. κ-carrageenan (KC), a linear polygalactose extracted from seaweed, has the functions of emulsification, thickening, gelling, and stabilization and is widely used in many fields [15,16]. Cao et al. added KC to sausages to improve gel properties [17]. Alavi et al. found that the curcumin loading capacity of whey protein isolate gel was significantly improved after adding KC [18].

Salt ions are another important factor that affects protein gelation [19]. A proper concentration of salt ions can increase the surface charge of the protein, thereby improving its solubility. In addition, studies have shown that salt ions can neutralize negatively charged amino acids, altering protein–polysaccharide interactions [20]. It is well known that NaCl is often added in the preparation process of many foods, so it is important to understand the behavior of protein gels in the presence of NaCl and that the electrostatic shielding of Na^+^ is beneficial to the formation of composite gels [21].

With this experiment, we aimed to explore the influence of Na^+^ concentration on the rheological, textural properties, and gel properties, as well as the water-holding capacity (WHC), of walnut protein isolate-κ-carrageenan (WNPI-KC) composite gels. Moreover, the interaction force and microstructure of WNPI-KC composite gels were also investigated to reveal the mechanism of Na^+^ on the gel and the bonding behavior of WNPI-KC composites. This research could reveal new methods for improving the gel properties of WNPI so as to expand the width and depth of the development and utilization of walnut protein resources.

## 2. Results and Discussion

### 2.1. Effect of Na^+^ Concentration on the Rheological and Texture Properties of WNPI-KC Composite Gel

#### 2.1.1. Rheological Properties

**Figure 1:**
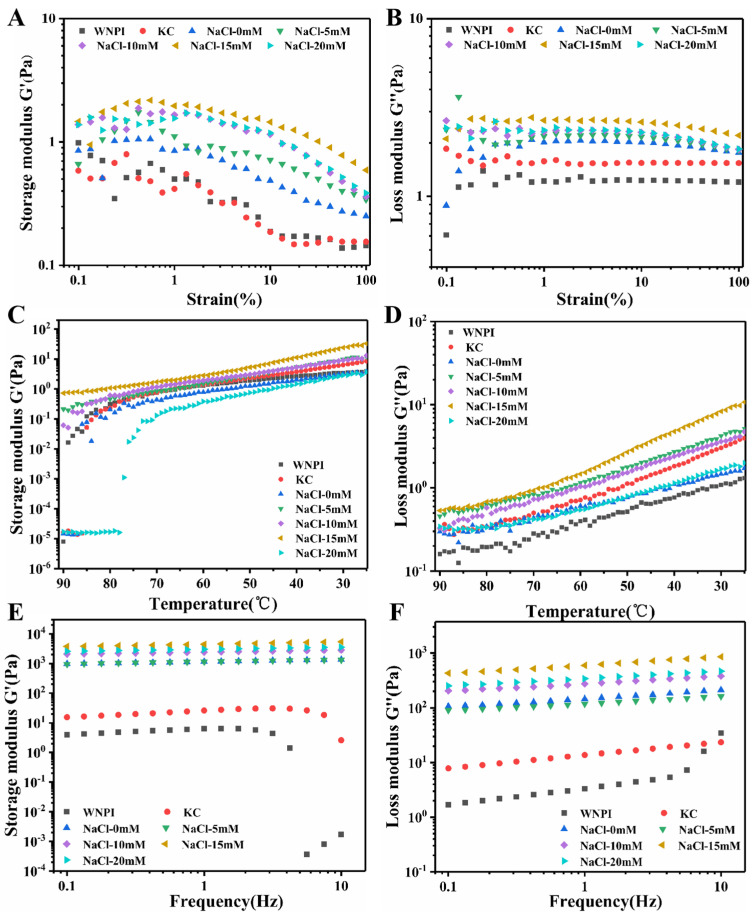
Rheological behaviors of WNPI-KC composite gel samples with NaCl. (**A**,**B**): Storage modulus (G′) and loss modulus (G″) of composite gel samples with NaCl during strain sweep; (**C**,**D**): G′ and G″ of the composite gel samples with NaCl during cooling process; (**E**,**F**): G′ and G″ of the composite gel samples with NaCl during frequency sweep.

Rheological behavior is often used to evaluate the gelling ability of proteins [22]. As shown in Figure 1A,B, by measuring the changes in G′ and G″ of the WNPI-KC composite gel with strain, the linear viscoelastic region was determined to be 1%, and the temperature and frequency of the WNPI-KC composite gel were scanned in this region. During the cooling stage, the G″ value of all gel samples was significantly lower than G′ at the same temperature, indicating that an elastic-based gel was formed (Figure 1C,D). With the increase in Na^+^ concentration from 0 to 15 mM, G′ and G″ of the composite gel increased, indicating that during gel formation, stronger molecular interactions occurred between WNPI and KC, which resulted in the enhanced network structure of the composite gel [23]. When Na^+^ concentration reached 20 mM, G′ and G″ values of the mixed gel decreased. Similar results were reported by Beck et al. [24]. In addition, G′ and G″ increased rapidly, indicating that the composite gel network continuously strengthened. Appropriate salt ion concentration was beneficial to protein aggregation and the formation of an elastic-dominant gel network, but excessive salt ion concentration led to excessive protein aggregation and was not conducive to the formation of gel.

As seen in Figure 1E,F, the modulus G′ and G″ values of all gel samples increased with increased angular frequency in the frequency scanning process in a frequency-dependent manner. At the same angular frequency, the G″ values were significantly lower than the same angular frequency of G′, indicating that the gel has strong elastic properties. The G′ and G″ of WNPI-KC composite gel increased from 0 to 15 mM with increased Na+ concentration, indicating that there was a stronger molecular interaction between WNPI and KC during gel formation. When Na^+^ concentration reached 20 mM, the G′ and G″ of WNPI-KC composite gel decreased. Wang et al. observed similar results, reporting that an appropriate Ca^2+^ concentration (0–200 mM) increased the G′ and G″ of rice glutelin-sugar beet pectin complex gels, but excessive Ca^2+^ (400 mM) decreased these values, which was not conducive to the formation of gel network [25].

#### 2.1.2. Textural Properties

Texture performance is an important indicator of gel properties. As shown in Table 1, the hardness, adhesiveness, gumminess, and chewiness of WNPI-KC gel increased slightly with increased NaCl concentration (0–15 mM), but there was no significant difference in springiness, cohesiveness, or resilience (*p* < 0.05). This is mainly because the appropriate concentration of Na^+^ reduced the repulsion between WNPI and KC and promoted the combination of WNPI and KC [26]. In addition, when the Na^+^ concentration reached 20 mM, the hardness, adhesion, and chewiness of the WNPI-KC gel decreased. This may be due to the excessive aggregation of proteins caused by the addition of 20 mM Na^+^ [27]. This was also consistent with the results of the microstructure. The dense gel network structure improved the texture properties of the gel [28].

### 2.2. Effect of Na^+^ on the Apparent Viscosity, Bond Strength, Water-Holding Capacity, and Thermal Stability of WNPI-KC Composite Gel

#### 2.2.1. Apparent Viscosity

**Figure 2:**
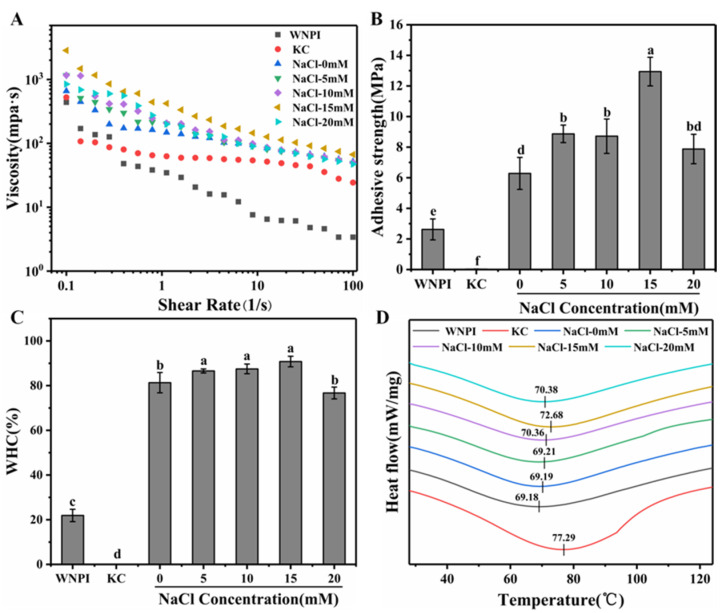
Apparent viscosity (**A**), Adhesive strength, (**B**) WHC (**C**), and DSC (**D**) of WNPI-KC composite gel samples with NaCl. Different letters on the bars indicate significant differences (*p* < 0.05).

The apparent viscosity of the WNPI-KC composite gel in the presence of NaCl is displayed in Figure 2A. As can be seen from the figure, 15 mM Na^+^ had the highest apparent viscosity. This behavior may be due to the fact that appropriate concentration of Na^+^ strengthened the hydrophobic interaction and the electrostatic interaction between WNPI and KC, and the high-molecular-weight polymers formed by the two may have caused higher apparent viscosity [29].

#### 2.2.2. Bond Strength

Bond strength is an important indicator for evaluating the adhesiveness of gels [30]. As shown in Figure 2B, the bonding strength of WNPI-KC composite was higher than that of a single protein and polysaccharide. After NaCl treatment, the bonding strength was further improved, with a maximum of 12.94 Mpa at an Na^+^ concentration of 15 mM. This might be due to the fact that Na^+^ promoted the unfolding of the protein molecular structure, increased the molecular force between WNPI and KC, and produced a good binding force, which is consistent with the results of apparent viscosity.

#### 2.2.3. Water-Holding Capacity (WHC)

WHC could reflect the ability of protein gel to retain water. As presented in Figure 2C, the WHC of the WNPI-KC gel was the highest at 15 mM Na^+^, at 90.18%. Na^+^ concentrations among 5–15 mM had no significant effect on the WHC of the composite gel (*p* > 0.05). It is possible that salt ions induced a dense gel structure. However, 20 mM Na^+^ might cause the gel network to be rough and irregular, resulting in a decrease in WHC. Zhang et al. found that the composite gel of wheat gluten protein and potato isolate protein had a dense network structure under low concentrations of Na^+^, whereas it was relatively loose under high ionic strength [31]. Studies have shown that a denser three-dimensional network structure better confines water in the gel system, thereby significantly enhancing its WHC [32]. 

#### 2.2.4. Thermal Stability

The effect of NaCl treatment on the thermal stability of the gel is presented in Figure 2D. The denaturation temperature of the gel without NaCl treatment was 69.19 °C. With an increase in Na^+^ concentration, the heat denaturation temperature gradually increased and reached a maximum value of 72.68 °C at 15 mM Na^+^. The rising trend of thermal denaturation temperature was reversed due to the salting-out effect when Na^+^ concentration reached 20 mM. Therefore, it can be inferred that the appropriate concentration of Na^+^ could improve the thermal stability of the composite gel. This might be due to the stronger gel structure and stronger intermolecular interaction of the composite gel induced by Na^+^, which was consistent with the findings from Zhou et al.

### 2.3. Effect of Na^+^ Concentration on the Moisture Distribution of WNPI-KC Composite Gel

**Figure 3:**
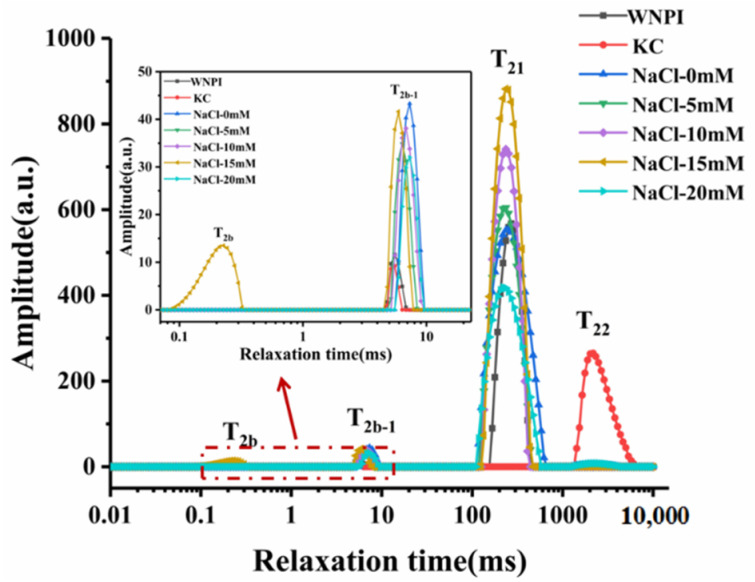
T_2_ relaxation times distribution of WNPI-KC composite gel samples with NaCl.

LF-NMR was used to characterize the moisture characteristics of the gel system, and the quality of the gel was reflected by the evaluation of the distribution of water molecules [33]. It can be seen from Figure 3 that the T_2_ curve possesses four separate peaks, which represent strongly bound water (T_2b_), weakly bound water (T_2b-1_), immobilized water (T_21_), and free water (T_22_). The concentration of Na^+^ at the T_2b_, T_2b-1_, T_21_, and T_22_ relaxation times of WNPI-KC composite gel and the corresponding peak areas, P_2b_, P_2b-1_, P_21_, and P_22_, are shown in Table 2. Substances with a shorter relaxation time were closer to water molecules than those with a longer relaxation time. The water content of different states in the gel system was determined by comparing the corresponding peak areas. It can be seen from the relaxation times of T_2b_ and T_2b-1_ after adding NaCl that the relaxation time showed a significantly shorter trend (*p* < 0.05). The T_2b-1_ relaxation time of the composite gel with 15 mM Na^+^ was the shortest, and the T_2b_ relaxation time appeared at the same time, revealing that the combination of WNPI and water molecules was tighter. After adding NaCl, the T_21_ relaxation time was shortened, and there was almost no change with different concentrations of NaCl (*p* > 0.05). The relaxation time of free water appeared at 20 mM Na^+^, which indicates that excessive Na^+^ concentration loosens the binding of water molecules and proteins. This result is consistent with WHC. It can be seen from the peak area that 15 mM Na^+^ reduced the immobilized water of the composite gel, increased the bound water, and migrated to the strongly bound water. This might be due to the enhanced binding ability of WNPI to water molecules by Na^+^ treatment.

### 2.4. Effect of Na^+^ on the Particle Size and Zeta Potential of WNPI-KC Composite Gel

**Figure 4:**
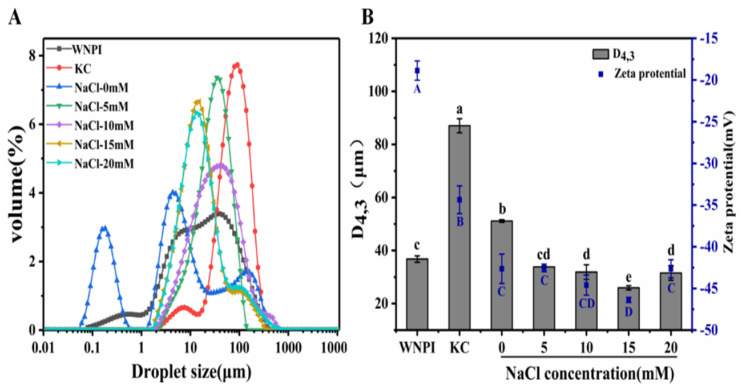
Effect of Na^+^ on the Particle size distribution (**A**), D[4,3] and zeta potential (**B**) of WNPIKC composite gel samples. Different letters in (**B**) indicate significant differences (*p* < 0.05).

The particle size distribution reflects the size and uniformity of the particles in the sample solution. D[4,3] represents the volume-weighted average particle diameter, which indicates the degree of protein aggregation. ζ-potential is a vital indicator for evaluating colloidal stability; the lower the absolute value of the potential, the lower the stability of the solution system [34]. The particle size distribution of all samples was roughly bimodal (Figure 4A), and D[4,3] of the composite gel was minimal at 15 mM NaCl (Figure 4B). This indicates that the appropriate concentration of Na^+^ ultrasound might cause the protein to unfold and reduce the particle size, thereby forming a much stabler and more uniform system, whereas an excessively high concentration of Na^+^ would cause the formation of protein aggregates, leading to an increase in the particle size. Compared with WNPI and KC alone, the absolute value of the potential of the WNPI-KC composite system increased significantly (*p* > 0.05) (Figure 4B). With the increase Na^+^ concentration, it first increased and then decreased, reaching a maximum at 15 mM (46.37 mV). This might be due to the fact that WNPI and KC both carried negative charges, which increased the negative charges in the composite system and electrostatic repulsion between the droplets [35]. NaCl also had a certain amount of negative charge, which resulted in an increase in negative charge on the surface of the droplet. When Na^+^ concentration was 20 mM, the protein reaggregated and shielded some charged groups, which increased the negative charge of WNPI-KC. The results show that the composite system had the smallest particle size and the highest absolute value of potential after treatment with 15 mM Na^+^, which effectively improved the stability of the composite system.

### 2.5. Effect of Na^+^ on the Structural Properties of WNPI-KC Composite Gel

#### 2.5.1. FTIR

**Figure 5:**
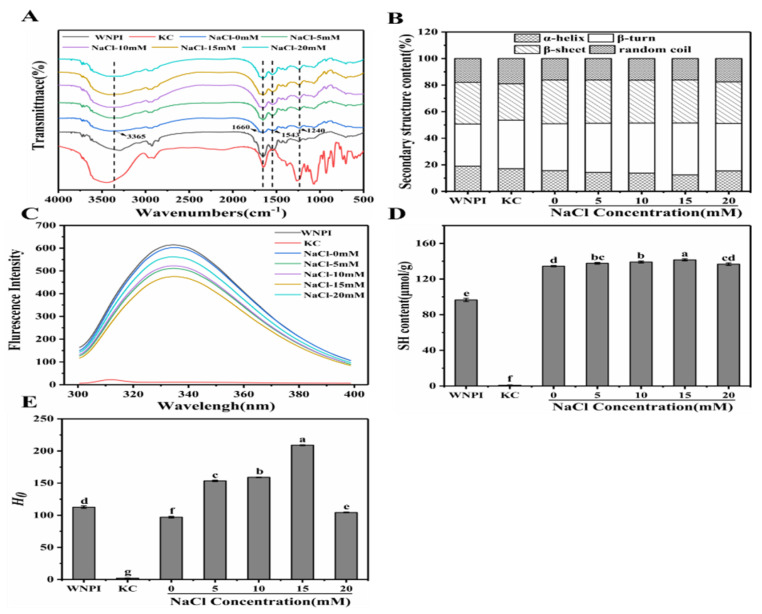
FTIR (**A**) structural content; (**B**) intrinsic fluorescence; (**C**) SH groups; (**D**) surface hydrophobicity; (**E**) WNPI-KC composite gel samples with NaCl. Different letters on the bars indicate significant differences (*p* < 0.05).

FTIR can be used to study the interaction between the various components in composite gel [36]. As shown in Figure 5A, the composite gel had several characteristic absorption peaks located at 3365 cm^−1^, 1660 cm^−1^, 1543 cm^−1^, and 1240 cm^−1^. No new peaks were observed in any of the samples, confirming that no new functional groups were generated by Na^+^ treatment. When the Na^+^ concentration was 0–15 mM, the broadband intensity from 3100 to 3500 cm^−1^ increased, indicating that the hydrogen bond strength was enhanced, but the high concentration of Na+ partially broke the hydrogen bond, resulting in a decrease in the broadband strength [37]. Similarly, peak intensities of amide I, amide II, and amide III bands were enhanced at low Na^+^ concentrations (0–15 mM), which might be another important reason why Na^+^ promoted the formation of a dense and stable network structure in WNPI-KC composite gel. The peak intensity decreased at a higher Na^+^ concentration (20 mM), which might be due to the weakened interaction between WNPI and KC, as well as the decreased thermal stability caused by changes in the protein during heating.

The secondary structure of WNPI was fitted according to the amide I region in FTIR [38] (Figure 5B). The α-helix content reduced from 15.64 (0 mM) to 12.39% (15 mM), whereas the β-sheet content increased from 35.23 to 39.02%. Studies have shown that an increase in β-sheet content is conducive to the formation of a dense and uniform gel network [39]. Zhao et al. also reported that the transformation of the α-helix to β-sheet of sweet potato protein was conducive to the formation of gel after adding salt ions [40]. In addition, with an increase in Na^+^ concentration (20 mM), the random coil content increased, indicating that the structure of the protein gradually became irregular.

#### 2.5.2. Fluorescence Spectroscopy

Fluorescence spectroscopy has been widely used to determine tertiary structural changes in proteins [41]. As described in Figure 5C, the fluorescence intensity of the KC solution was close to 0, and the other samples all had maximum fluorescence emissions at 335 nm. Compared with WNPI alone, the fluorescence intensity of WNPI-KC composite solution was significantly reduced. As the concentration of Na^+^ increased, the fluorescence intensity first increased and then decreased reaching the lowest value at 15 mM (475). This might be due to the fact that Na+ accelerated the WNPI molecule-deployable structure and the chromophore was exposed to the solvent, which changed the polarity of the environment and reduced the fluorescence intensity [42]. Pallarès et al. revealed that the decrease in fluorescence intensity might be due to the tight tertiary conformation in WNPI shielding tryptophan residues [43]. The increased fluorescence intensity at high concentrations of Na^+^ (20 mM) might be due to the weakening of the interaction between WNPI and KC.

#### 2.5.3. Sulfhydryl Content

Sulfhydryl content can reveal changes in protein conformation and the degree of protein denaturation, which play an important role in the functional properties of the protein. With an increase in Na^+^ concentration, the content of -SH in the composite solution increased gradually, and the highest sulfhydryl content was 141.54 μmol/g at 15 mM (Figure 5D). Na^+^-induced protein unfolding was beneficial to the exposure of internal -SH groups, resulting in an increase in -SH content. Liu et al. reported that the disulfide bonds induced by sulfhydryl groups facilitated the formation of a gel network during thermal gelation [44]. In addition, the increase in free sulfhydryl groups enhanced the hydrophobic interaction between WNPI molecules and induced the composite system to form a stable gel. However, 20 mM Na^+^ caused protein molecules to aggregate, and sulfhydryl groups were buried, resulting in a decrease in their content.

#### 2.5.4. Surface Hydrophobicity

Surface hydrophobicity can reflect changes in protein structural properties [45]. The surface hydrophobicity showed an increasing trend when the Na^+^ concentration increased from 0 mM to 15 mM and decreased at 20 mM Na^+^ (Figure 5E). The maximum surface hydrophobicity of the composite solution was 209.00 at 15 mM Na^+^. This may be because Na+ promotes the unfolding of the WNPI structure, exposing the hydrophobic group originally embedded in WNPI on the surface. However, excessive Na^+^ (20 mM) caused salting out, and the hydrophobic groups and -SH groups would be hidden, which might affect the gel performance of the protein [46].

#### 2.5.5. Microstructure

**Figure 6:**
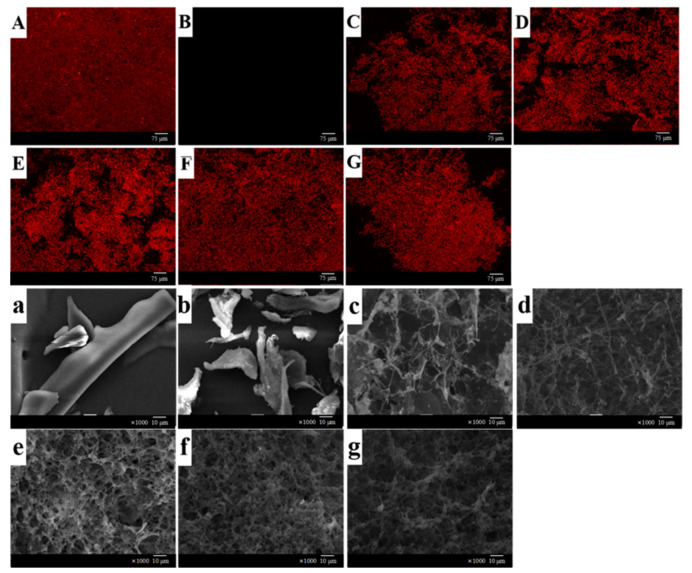
CLSM and SEM images of WNPI-KC composite gel samples with NaCl. (**A**–**G**, **a**–**g**) WNPI, KC, NaCl-0 mM, NaCl-5 mM, NaCl-10 mM, NaCl-15 mM, and NaCl-20 mM, respectively.

The influence of Na^+^ concentration on the three-dimensional network structure of WNPI-KC composite gel was directly observed by laser scanning confocal microscopy. WNPI was marked as red by rhodamine B, and KC was marked as dark black (Figure 6A–G). At low concentrations of Na^+^ (0–15 mM), the protein molecules gradually unfolded, and the microstructure of the WNPI-KC gel gradually became homogeneous, indicating that the interaction between WNPI and KC was enhanced. However, 20 mM Na^+^ aggregated protein molecules, suggesting that the microstructure was destroyed by excessive Na^+^. Zhang et al. reported that an appropriate concentration of Na^+^ promoted the cross linking and polymerization of wheat gluten protein and potato isolate protein molecules, forming a uniform and dense gel network; however, with a continuous increase in Na^+^, the network structure of the gel was destroyed.

The morphology of the WNPI-KC gel was further observed by scanning electron microscopy. The single protein had a sheet-like structure, and the polysaccharide had a rod-like structure (Figure 6a–g). The complex gel network without salt ions was relatively rough and had large gaps. With Na^+^ concentration increased from 5 mM to 15 mM, the gel became dense with smaller pores, but at higher Na^+^ concentrations, the gel matrix presented a concave–convex surface with many holes, which is consistent with the observation results of CLSM. Yang et al. also found that an appropriate concentration of Na^+^ caused the protein form a uniform and dense network structure during heating. Excessive salt ions disrupted the balance and formed protein aggregation, resulting in a rough and uneven morphology, which might lead to reduced WHC and textural properties [47].

### 2.6. Effect of Na^+^ on the Intermolecular Force of WNPI-KC Composite Gel

**Figure 7:**
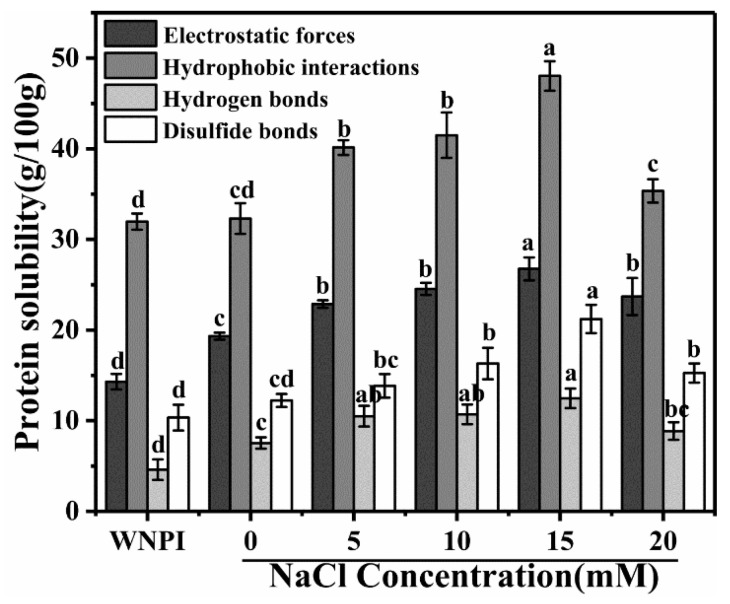
Molecular interaction forces of WNPI-KC composite gel samples with NaCl. Different letters (**a**–**d**) on differently colored bars indicate significant differences (*p* < 0.05).

The intermolecular forces were determined by measuring the solubility of WNPI-KC gel in different solutions [48]. It can be seen from Figure 7 that the main interaction force in the composite gel was hydrophobic interaction, which is consistent with the research results of Xia et al. [49]. The low concentration of Na^+^ (5–15 mM) enhanced the electrostatic interaction, but as the Na^+^ concentration further increased, the electrostatic interaction weakened. This may be due to the fact that a moderate concentration of NaCl can reduce the electrostatic repulsion between proteins and polysaccharides, thereby narrowing the distance between the two and promoting the formation of aggregates. In addition, the salt bridge formed by Na^+^ and protein molecules also reduced the electrostatic repulsion. However, excessive Na^+^ caused the protein particles aggregate and precipitate quickly, thereby reducing the electrostatic repulsion. Compared with composite gels that did not contain Na^+^, 15 mM Na^+^ enhanced hydrophobic interactions. This indicates that Na^+^ exposed the hydrophobic groups inside the protein molecules. However, WNPI might form irregular aggregates at 20 mM NaCl, reducing hydrophobic interactions. When the concentration of NaCl is 15mM, the hydrogen bonding effect is the strongest, which may be due to the fact that Na+ promoted the interaction between WNPI and water molecules [50]. The strength of disulfide bonds showed the same trend as that of hydrogen bonds. It is well known that sulfhydryl groups can induce the generation of disulfide bonds and promote the cross linking of proteins and polysaccharides during heating. The concentration of Na^+^ at 15 mM induced the production of more –SH, so the content of disulfide bonds increased.

### 2.7. Effect of Na^+^ on the Schematic Mechanism of WNPI-KC Composite Gel

**Figure 8:**
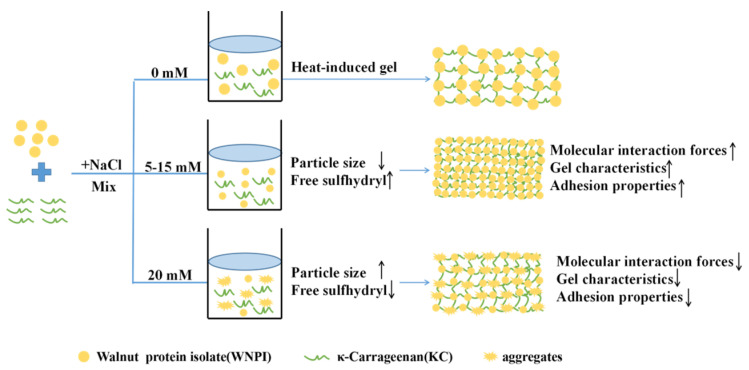
Schematic mechanism of WNPI-KC composite gel samples with NaCl.

Based on the above research results, the gelling mechanisms of different concentrations of NaCl on WNPI-KC composite are shown in Figure 8. In the absence of NaCl, WNPI-KC composite gel did not show good gel strength, so the bond strength was low. At 15 mM NaCl, WNPI-KC composite gel had the best gel properties. This is because the combination of electrostatic shielding and ion bridging cause the protein molecule to unfold, and more sulfhydryl groups and hydrophobic groups in the protein molecule were exposed and enhanced the interaction between WNPI and KC, which promoted the ordered aggregation of the composite system, forming an ordered network structure with the best gel performance. However, salting out occurred at 20 mM NaCl, which led to excessive aggregation of the composite system, resulting in a rough and irregular structure in the gel system, so it had poor gel strength. In conclusion, 15 mM NaCl improved the gel properties of the WNPI-KC composite system. 

## 3. Conclusions

In this study, we explored the influence of NaCl concentration on the rheological, structural, and gelling properties of WNPI-KC composite gel. The results showed that an appropriate concentration of Na^+^ (15 mM) significantly increased the free sulfhydryl content and surface hydrophobicity of WNPI-KC and promoted the transformation of its structure from α-helix to β-sheet, which was beneficial to promote the orderly aggregation of the composite system and formation of an orderly network structure. A concentration of 15 mM Na^+^ also enhanced the interaction force between WNPI and KC; thus, the cohesiveness, rheological properties, texture properties, and WHC of the composite gel were significantly improved. However, excessive Na^+^ (20 mM) led to excessive aggregation of the composite system, resulting in irregular gel structure and damage to the gel and bond strength. With this experiment, we constructed a new type of salt-ion-regulated walnut protein gel system, which could represent a new method for improving the gel properties of WNPI. It could also provide technical support for its application in the food industry and other related industries. Further research is needed concerning the mechanism of action and practical application scenarios.

## 4. Materials and Methods

### 4.1. Materials

Walnuts were obtained from a local market in Wuhan (Hubei, China). κ-carrageenan was obtained from Henan Chinuo Food Ingredients Co., Ltd. (Henan, China). Other chemicals were obtained from Sinopharm Chemical Reagent Co., Ltd. (Shanghai, China).

### 4.2. Preparation of WNPI and WNPI-KC Composite Gels

WNPI was prepared according to the method described in a previous study [51]. A given amount of WNPI, KC, and NaCl was mixed in deionized water and stirred magnetically at 25 °C for 2 h. The final concentration of WNPI and KC in the composite solutions was 5% (*w*/*v*) and 0.5% (*w*/*v*), respectively. The Na^+^ concentrations were 0, 5, 10, 15, and 20 mmol/L. Then, the composite solution was heated in a 90 °C water bath for 30 min, immediately cooled, and then kept at 4 °C for 12 h to prepare WNPI-KC composite gel.

### 4.3. Rheological Measurement

The dynamic rheological properties of the composite gels were determined with an Anton Paar rheometer (Thermo Fisher Scientific, Karlsruhe, Germany) consisting of a parallel-plate geometry (40 mm diameter, 1 mm gap) [52]. The parameters of the viscosity scan were: shear rate, 0.01–100 s^−1^; temperature, 25 °C. The parameters of the amplitude sweep were: temperature, 25 °C; frequency, 1 Hz; strain, 0.1–100%. The parameters of the temperature scan were: frequency, 1 Hz; strain, 1%; rate, 5 °C/min; temperature, 25–90 °C (heating) or 90–25 °C (cooling). The parameters of the frequency sweep were: strain, 10%; angular frequency, 0.1–10 rad/s.

### 4.4. Texture Analysis

The textural properties (hardness, adhesiveness, springiness, cohesiveness, gumminess, chewiness, and resilience) of WNPI-KC composite gels (15 mm height and 30 mm diameter) were determined using a texture analyzer (Stable Micro System, Godalming, UK) [53]. A P/0.5 cylindrical probe and TPA mode were selected, and the test parameters were set as follows: compression rate, 30%; trigger force, 5 g; pre-test speed, test speed, and post-test speed: 1.0 mm/s, 0.5 mm/s, and 1.0 mm/s, respectively.

### 4.5. Determination of Bond Strength

Three samples of the same size were glued together with a gel sample using corrugated cardboard as a material. The bonding strength was measured with an electromechanically universal testing machine with a tensile speed of 10 mm/min. The average value of each sample was taken, and the corresponding test was carried out six times [54].

### 4.6. Water-Holding Capacity (WHC)

An amount of 5 g gel sample was placed in a centrifuge tube and centrifuged at 8000 r/min for 10 min. WHC is expressed as the ratio of the gel weight after centrifugation to the gel weight before centrifugation [55].

### 4.7. Thermal Stability

A Micro-DSCIII differential scanning calorimeter (DSC) (TA Instruments, New Castle, DE, USA) was used to determine the thermal stability of the composite gel [56]. A given amount of lyophilized gel powder was added to the crucible, and then the heating rate and scanning temperature were set to 10 °C/min and 25–125 °C, respectively. 

### 4.8. Low-Field Nuclear Magnetic Resonance (LF-NMR)

The moisture distribution of the composite gel was determined using an LF-NMR analyzer (MesoMR, Niumag Electronic Technology Co., Ltd., Shanghai, China) as described by Zhang et al., with minor modifications [57]. The number of scans, echo time, and waiting time were set to 4, 0.5, and 5000 ms, respectively. A total of 4000 echoes were collected for analysis. DLX software was used to analyze the results through the corresponding relative signal amplitude.

### 4.9. Particle Size and Potential

The D[3,4] of the composite solutions was measured with a Malvern 2000 instrument (Malvern Instruments, Zetasizer Nano SZ, Malvern, UK) with a pump speed of 2000 r/min. The zeta potential of the composite solutions was measured using a Nano-ZS zetasizer(Malvern Instruments, Zetasizer Nano SZ, Malvern, UK) after 50-fold dilution in deionized water at 25 °C [58].

### 4.10. Fourier Transform Infrared (FTIR) Spectroscopy

The freeze-dried gel sample was scanned 64 times in the range of 4000 cm^−1^ to 400 cm^−1^ with a resolution of 4 cm^−1^ using an FTIR spectrometer [59]. The changes in secondary structure of amide I bonds in the 1600–1700 cm^−1^ range were analyzed by Omnic and PeakFit software. The content of each secondary structure was calculated by absorption peak area.

### 4.11. Fluorescence Spectroscopy

The fluorescence spectra of composite solutions (1 mg/mL) were measured by a fluorescence spectrofluorometer. The parameters were set as follows: excitation wavelength, 280 nm; scanning range, 300–400 nm; scanning speed, 1200 nm/min; slit width, 5 nm.

### 4.12. SH Content

A volume of 4 mL of WNPI-KC composite solution (1 mg/mL) was mixed with 40 μL DTNB solution, and the reaction was incubated at 25 °C for 30 min in the dark [60]. The calculation method was as follows:

(1) $$Sulfhydryl\;(\mu \mathrm{mol}/100\;\mathrm{mg})=\frac{A_{412}{\times D\times 10}^{5}}{{13\text{,}600\times C}_{pr}}$$

where

*A*_412_ represents the absorbance at 412 nm;
*D* represents the dilution factor; and
*C_p_r* represents the sample concentration (mg/mL).

### 4.13. Surface Hydrophobicity

The surface hydrophobicity of the samples was measured according to the method of Wang et al., with slight modifications [61]. The samples treated with different concentrations of NaCl were diluted into solutions with a concentration of 0.1–0.5 mg/mL, and 20 μL of 8 mmol/L ANS was added to 4 mL of different concentrations of solution. The reaction was performed in the dark for 20 min at 25 °C, and then the fluorescence intensity was measured with a fluorescence spectrophotometer. The measurement parameters were set as: excitation wavelength, 390 nm; emission wavelength, 470 nm; slit, 5 nm; scanning speed, 240 nm/min. Finally, the fluorescence intensity was plotted versus protein concentration, and its initial slope was used as an index of protein surface hydrophobicity.

### 4.14. Microstructure

The CLSM of the composite gels was observed using a laser confocal microscope(Tecnai G2 Spirit BIOTWIN, FEI, Rochester, NY, USA). Before observation, the composite solution (20 μL/mL) was stained with 0.02% rhodamine B reagent. The surface morphology of the WNPI-KC gel was observed at 1000 times magnification using SEM as described by Feng et al. [62].

### 4.15. Molecular Interaction Forces

The intermolecular forces during WNPI-XG gel formation were measured according to the method described by Sun et al. [63]. An amount of 0.5 g of gel samples was mixed with ultrapure water (pH 8.0), 0.086 M Tris-0.09 M glycine-4 mM-Na2EDTA (pH 8.0), 0.5% SDS, 8 M urea, and 2% β-mercaptoethanol. After 20 min of reaction, samples were centrifuged (5000× *g*, 15 min) and the supernatant (8000× *g*, 10 min) was obtained, and the solubility of the protein in the supernatant was calculated according to the Biuret method.

### 4.16. Statistical Analysis

Analysis of variance (ANOVA) and Tukey’s test were performed on the data using SPSS 25.0, and Origin 2017 was used for graph drawing. Differences were considered statistically significant when *p* < 0.05. Each experiment was performed in triplicate, and the results are expressed as the mean ± standard deviation.

## Figures and Tables

**Table 1:** Textural properties of WNPI-KC composite gel samples with NaCl.

	Hardness	Adhesiveness	Springiness	Cohesiveness	Gumminess	Chewiness	Resilience
WNPI	9.05 ± 1.06 ^e^	−3.71 ± 0.16 ^d^	0.55 ± 0.05 ^b^	0.38 ± 0.03 ^c^	3.48 ± 0.70 ^e^	1.92 ± 0.55 ^e^	0.32 ± 0.01 ^b^
NaCl-0 mM	47.94 ± 2.06 ^d^	−5.80 ± 0.69 ^c^	0.86 ± 0.05 ^a^	0.52 ± 0.15 ^bc^	24.07 ± 1.58 ^d^	21.60 ± 2.12 ^d^	0.51 ± 0.08 ^a^
NaCl-5 mM	61.91 ± 0.14 ^c^	−7.82 ± 0.90 ^b^	0.91 ± 0.04 ^a^	0.65 ± 0.11 ^ab^	41.19 ± 2.65 ^c^	37.71 ± 2.44 ^c^	0.53 ± 0.09 ^a^
NaCl-10 mM	70.83 ± 0.60 ^b^	−7.92 ± 1.01 ^b^	0.91 ± 0.00 ^a^	0.76 ± 0.03 ^a^	53.65 ± 2.21 ^b^	48.54 ± 2.1 ^b^	0.51 ± 0.04 ^a^
NaCl-15 mM	96.18 ± 3.85 ^a^	−10.79 ± 1.85 ^a^	0.92 ± 0.01 ^a^	0.76 ± 0.05 ^a^	70.38 ± 2.72 ^a^	64.86 ± 1.89 ^a^	0.50 ± 0.03 ^a^
NaCl-20 mM	76.29 ± 1.07 ^b^	−6.99 ± 0.52b ^c^	0.92 ± 0.04 ^a^	0.69 ± 0.16 ^ab^	52.30 ± 2.97 ^b^	48.47 ± 2.24 ^b^	0.52 ± 0.07 ^a^

Different letters in the same column indicate significant differences (*p* < 0.05).

**Table 2:** T_2_ relaxation times and relative peak areas of WNPI-KC composite gel samples with NaCl.

	T_2b_(ms)	T_2b-1_(ms)	T_21_(ms)	T_22_(ms)	P_2b_(%)	P_2b-1_(%)	P_21_(%)	P_22_(%)
WNPI	--	5.29 ± 0.21 ^e^	264.45 ± 10.47 ^a^	--	--	0.71 ± 0.07 ^d^	99.29 ± 0.07 ^a^	--
KC	--	5.18 ± 0.36 ^e^	--	2025.50 ± 0.00 ^c^	--	0.41 ± 0.02 ^e^	--	99.59 ± 0.02 ^a^
NaCl-0 mM	--	7.32 ± 0.00 ^a^	241.07 ± 9.77 ^b^	2171.12 ± 0.00 ^b^	--	2.21 ± 0.00 ^a^	97.18 ± 0.16 ^c^	0.62 ± 0.16 ^b^
NaCl-5 mM	--	6.37 ± 0.00 ^c^	224.90 ± 9.12 ^c^	--	--	1.92 ± 0.07 ^b^	98.08 ± 0.07 ^b^	--
NaCl-10 mM	--	6.83 ± 0.00 ^b^	235.43 ± 0.00 ^bc^	--	--	2.13 ± 0.05 ^a^	97.87 ± 0.05 ^b^	--
NaCl-15 mM	0.22 ± 0.01 ^a^	5.94 ± 0.00 ^d^	235.43 ± 0.00 ^bc^	--	1.46 ± 0.18 ^a^	1.69 ± 0.06 ^c^	96.85 ± 0.12 ^c^	--
NaCl-20 mM	--	6.99 ± 0.28 ^ab^	230.17 ± 9.12 ^bc^	2327.20 ± 0.00 ^a^	--	2.17 ± 0.08 ^a^	96.75 ± 0.50 ^c^	1.07 ± 0.43 ^b^

Different letters in the same column indicate significant differences (*p* < 0.05).

## Data Availability

Not applicable.

## References

[1] Chen Y., Pei H., Dai Q., Zhang C., Kong X., Hua Y. (2021). Raw walnut kernel: A natural source for dietary proteases and bioactive proteins. Food Chem..

[2] Alasalvar C., Salvadó J.-S., Ros E. (2020). Bioactives and health benefits of nuts and dried fruits. Food Chem..

[3] Ros E., Núñez I., Pérez-Heras A., Serra M., Gilabert R., Casals E., Deulofeu R. (2004). A walnut diet improves endothelial function in hypercholesterolemic subjects: A randomized crossover trial. Circulation.

[4] Zhu Z., Zhu W., Yi J., Liu N., Cao Y., Lu J., Decker E.A., McClements D.J. (2018). Effects of sonication on the physicochemical and functional properties of walnut protein isolate. Food Res. Int..

[5] Moghadam M., Salami M., Mohammadian M., Emam-Djomeh Z., Jahanbani R., Moosavi-Movahedi A.A. (2020). Physicochemical and bio-functional properties of walnut proteins as affected by trypsin-mediated hydrolysis. Food Biosci..

[6] Sze-Tao K., Sathe S. (2000). Walnuts (*Juglans regia* L.): Proximate composition, protein solubility, protein amino acid composition and protein in vitro digestibility. J. Sci. Food Agric..

[7] Yan C., Zhou Z. (2021). Solubility and emulsifying properties of phosphorylated walnut protein isolate extracted by sodium trimetaphosphate. LWT.

[8] Kong X., Zhang L., Song W., Zhang C., Li X. (2021). Separation, identification and molecular binding mechanism of dipeptidyl peptidase IV inhibitory peptides derived from walnut (*Juglans regia* l.) protein. Food Chem..

[9] Shi A., Jiao B., Liu H., Zhu S., Shen M., Feng X., Hu H., Liu L., Faisal S., Wang Q. (2018). Effects of proteolysis and transglutaminase crosslinking on physicochemical characteristics of walnut protein isolate. LWT.

[10] Cai Y., Deng X., Liu T., Zhao M., Zhao Q., Chen S. (2018). Effect of xanthan gum on walnut protein/xanthan gum mixtures, interfacial adsorption, and emulsion properties. Food Hydrocoll..

[11] Zheng H., Beamer S.K., Matak K.E., Jaczynski J. (2019). Effect of κ-carrageenan on gelation and gel characteristics of Antarctic krill (*Euphausia superba*) protein isolated with isoelectric solubilization/precipitation. Food Chem..

[12] Le X.T., Rioux L.E., Turgeon S.L. (2017). Formation and functional properties of protein-polysaccharide electrostatic hydrogels in comparison to protein or polysaccharide hydrogels. Adv. Colloid Interface Sci..

[13] Zhao H., Chen J., Hemar Y., Cui B. (2020). Improvement of the rheological and textural properties of calcium sulfate-induced soy protein isolate gels by the incorporation of different polysaccharides. Food Chem..

[14] Zhuang X., Wang L., Jiang X., Chen Y., Zhou G. (2021). Insight into the mechanism of myofibrillar protein gel influenced by konjac glucomannan: Moisture stability and phase separation behavior. Food Chem..

[15] Yemenicioğlu A., Farris S., Turkyilmaz M., Gulec S. (2020). A review of current and future food applications of natural hydrocolloids. Int. J. Food Sci. Technol..

[16] Zia K.M., Tabasum S., Nasif M., Sultan N., Aslam N., Noreen A., Zuber M. (2017). A review on synthesis, properties and applications of natural polymer based carrageenan blends and composites. Int. J. Biol. Macromol..

[17] Cao C., Feng Y., Kong B., Xia X., Liu M., Chen J., Liu Q. (2021). Textural and gel properties of frankfurters as influenced by various κ-carrageenan incorporation methods. Meat Sci..

[18] Alavi F., Emam-Djomeh Z., Yarmand M.S., Salami M., Momen S., Moosavi-Movahedi A.A. (2018). Cold gelation of curcumin loaded whey protein aggregates mixed with k-carrageenan: Impact of gel microstructure on the gastrointestinal fate of curcumin. Food Hydrocoll..

[19] Xia W., Ma L., Chen X., Li X., Zhang Y. (2018). Physicochemical and structural properties of composite gels prepared with myofibrillar protein and lecithin at various ionic strengths. Food Hydrocoll..

[20] Xiao Y., Kang S., Liu Y., Guo X., Li M., Xu H. (2021). Effect and mechanism of calcium ions on the gelation properties of cellulose nanocrystals-whey protein isolate composite gels. Food Hydrocoll..

[21] Yang Z., Campo L., Gilbert E.P., Knott R., Cheng L., Storer B., Lin X., Luo L., Patole S., Hemar Y. (2022). Effect of Na+ and CaCl_2_ concentration on the rheological and structural characteristics of thermally-induced quinoa protein gels. Food Hydrocoll..

[22] Savadkoohi S., Farahnaky A. (2012). Dynamic rheological and thermal study of the heat-induced gelation of tomato-seed proteins. J. Food Eng..

[23] Zhou F., Pan M., Liu Y., Guo N., Zhang Q., Wang J. (2020). Effects on Na+ on the cold gelation between a low-methoxyl pectin extracted from Premna microphylla turcz and soy protein isolate. Food Hydrocoll..

[24] Beck M., Jekle M., Becker T. (2012). Impact of sodium chloride on wheat flour dough for yeast-leavened products. I. Rheological attributes. J. Sci. Food Agric..

[25] Wang Y., Yang Q., Li-Sha Y., Chen H. (2020). Structural, gelation properties and microstructure of rice glutelin/sugar beet pectin composite gels: Effects of ionic strengths. Food Chem..

[26] Zhang Y., Tang C., Wen Q., Yang X., Li L., Deng W. (2010). Thermal aggregation and gelation of kidney bean (*Phaseolus vulgaris* L.) protein isolate at pH 2.0: Influence of ionic strength. Food Hydrocoll..

[27] Wang L., Fogliano V., Heising J., Meulenbroeks E., Dekker M. (2020). Volatile antimicrobial absorption in food gel depends on the food matrix characteristics. Food Hydrocoll..

[28] Jin H., Chen J., Zhang J., Sheng L. (2021). Impact of phosphates on heat-induced egg white gel properties: Texture, water state, micro-rheology and microstructure. Food Hydrocoll..

[29] Li S., Wang K., Huang Q., Geng F. (2021). Microwave pretreatment enhanced the properties of ovalbumin-inulin-oil emulsion gels and improved the storage stability of pomegranate seed oil. Food Hydrocoll..

[30] Huang W., Sun X. (2000). Adhesive properties of soy proteins modified by urea and guanidine hydrochloride. J. Am. Oil Chem. Soc..

[31] Zhang Z., Chen X., Liu X., Liu W., Liu Q., Huang J., Zhang L., Hu H. (2022). Effect of salt ions on mixed gels of wheat gluten protein and potato isolate protein. LWT.

[32] Farjami T., Madadlou A., Labbafi M. (2016). Modulating the textural characteristics of whey protein nanofibril gels with different concentrations of calcium chloride. J. Dairy Res..

[33] Panchal B., Truong T., Prakash S., Bansal N., Bhandari B. (2020). Effect of water content, droplet size, and gelation on fat phase transition and water mobility in water-in-milk fat emulsions. Food Chem..

[34] Zhang X., Zhang S., Zhong M., Qi B., Li Y. (2022). Soy and whey protein isolate mixture/calcium chloride thermally induced emulsion gels: Rheological properties and digestive characteristics. Food Chem..

[35] Griffin K., Khouryieh H. (2020). Influence of electrostatic interactions on the formation and stability of multilayer fish oil-in-water emulsions stabilized by whey protein-xanthan-locust bean complexes. J. Food Eng..

[36] Kyomugasho C., Christiaens S., Shpigelman A., Van Loey A.M., Hendrickx M.E. (2015). FT-IR spectroscopy, a reliable method for routine analysis of the degree of methylesterification of pectin in different fruit- and vegetable-based matrices. Food Chem..

[37] Bhargava N., Mor R.S., Kumar K., Sharanagat V.S. (2021). Advances in application of ultrasound in food processing: A review. Ultrason. Sonochem..

[38] Barth A. (2007). Infrared spectroscopy of proteins. Biochim. Biophys. Acta.

[39] Li Y., Wang Q., Guo L., Ho H., Wang B., Sun J., Xu X., Huang M. (2019). Effects of ultrafine comminution treatment on gelling properties of myofibrillar proteins from chicken breast. Food Hydrocoll..

[40] Zhao Z., Mu T., Zhang M., Richel A. (2018). Effect of salts combined with high hydrostatic pressure on structure and gelation properties of sweet potato protein. LWT Food Sci. Technol..

[41] Sheng L., Su P., Han K., Chen J., Cao A., Zhang Z., Jin Y., Ma M. (2017). Synthesis and structural characterization of lysozyme-pullulan conjugates obtained by the Maillard reaction. Food Hydrocoll..

[42] Xiong W.F., Wang Y.T., Zhang C.L. (2016). High intensity ultrasound modified ovalbumin: Structure, interface and gelation properties. Ultrason. Sonochem..

[43] Pallarès I., Vendrell J., Avilés F.X., Ventura S. (2004). Amyloid fibril formation by a partially structured intermediate state of α-chymotrypsin. J. Mol. Biol..

[44] Liu K., Li Q., Pan L., Qian X., Zhang H., Zha X., Luo J. (2017). The effects of lotus root amylopectin on the formation of whey protein isolate gels. Carbohydr. Polym..

[45] Hu H., Li-Chan E.C.Y., Wan L., Tian M., Pan S. (2013). The effect of high intensity ultrasonic pre-treatment on the properties of soybean protein isolate gel induced by calcium sulfate. Food Hydrocoll..

[46] Yang Q., Wang Y., Li-Sha Y., Chen H. (2021). Physicochemical, structural and gelation properties of arachin-basil seed gum composite gels: Effects of salt types and concentrations. Food Hydrocoll..

[47] Wang W., Shen M., Liu S., Jiang L., Song Q., Xie J. (2018). Gel properties and interactions of Mesona blumes polysaccharide-soy protein isolates mixed gel: The effect of salt addition. Carbohydr. Polym..

[48] Diao X., Guan H., Zhao X., Diao X., Kong B. (2016). Physicochemical and structural properties of composite gels prepared with myofibrillar protein and lard diacylglycerols. Meat Sci..

[49] Xia Q., Gu M., Liu J., Niu Y., Yu L. (2018). Novel composite gels of gelatin and soluble dietary fiber from black bean coats with interpenetrating polymer networks. Food Hydrocoll..

[50] Sun C., Chen S., Dai L., Gao Y. (2017). Structural characterization and formation mechanism of zein-propylene glycol alginate binary complex induced by calcium ions. Food Res. Int..

[51] Lei Y., Gao S., Xiang X., Li X., Yu X., Li S. (2021). Physicochemical, structural and adhesion properties of walnut protein isolate-xanthan gum composite adhesives using walnut protein modified by ethanol. Int. J. Biol. Macromol..

[52] Wang X., Zeng M., Qin F., Adhikari B., He Z., Chen J. (2018). Enhanced CaSO_4_-induced gelation properties of soy protein isolate emulsion by pre-aggregation. Food Chem..

[53] Zhang X., Wang W., Wang Y., Wang Y., Wang X., Gao G., Chen G., Liu A. (2018). Effects of nanofiber cellulose on functional properties of heat-induced chicken salt-soluble protein gel enhanced with microbial transglutaminase. Food Hydrocoll..

[54] Chen H., Xu Z., Mo J., Lyu Y., Shen X. (2017). Effect of guar gum on adhesion properties of soybean protein isolate onto porcine bones. Int. J. Adhes. Adhes..

[55] Tang C.H., Chen L., Foegeding E.A. (2011). Mechanical and water-holding properties and microstructures of soy protein isolate emulsion gels induced by CaCl_2_, glucono-δ-lactone (GDL), and transglutaminase: Influence of thermal Treatments before and/or after emulsification. J. Agric. Food Chem..

[56] Tanger C., Engel J., Kulozik U. (2020). Influence of extraction conditions on the conformational alteration of pea protein extracted from pea flour. Food Hydrocoll..

[57] Zhang B., Yao H., Qi H., Ying X. (2020). Cryoprotective characteristics of different sugar alcohols on peeled Pacific white shrimp (*Litopenaeus vannamei*) during frozen storage and their possible mechanisms of action. Int. J. Food Prop..

[58] Paraskevi Z., Spyridon M., Anastasia B., Sophia G.A. (2020). Preparation, physicochemical properties, and in vitro toxicity towards cancer cells of novel types of arsonoliposomes. Pharmaceutics.

[59] Li P., Sun Z., Ma M., Jin Y., Sheng L. (2018). Effect of microwave-assisted phosphorylation modification on the structural and foaming properties of egg white powder. LWT Food Sci. Technol..

[60] Liu K., Li Q., Zha X., Pan L., Bao L., Zhang H., Luo J. (2019). Effects of calcium or sodium ions on the properties of whey protein isolate-lotus root amylopectin composite gel. Food Hydrocoll..

[61] Wang K., Luo S., Zhong X., Cai J., Jiang S., Zheng Z. (2016). Effects of partial hydrolysis and subsequent cross-linking on wheat gluten physicochemical properties and structure. Food Chem..

[62] Feng Z., Dou W., Alaxi S., Niu Y., Yu L. (2017). Modified soluble dietary fiber from black bean coats with its rheological and bile acid binding properties. Food Hydrocoll..

[63] Sun X.D., Arntfield S.D. (2012). Molecular forces involved in heat-induced pea protein gelation: Effects of various reagents on the rheological properties of salt extracted pea protein gels. Food Hydrocoll..

